# Qualitative insights into planning implementation of FeNO-guided asthma management in primary care

**DOI:** 10.1038/s41533-025-00418-w

**Published:** 2025-03-20

**Authors:** G. Lewis, K. Morton, M. Santillo, L. Yardley, K. Wang, B. Ainsworth, S. Tonkin-Crine

**Affiliations:** 1 University of Bristol, Bristol, UK; 2 University of York, York, UK; 3 University of Oxford, Oxford, UK; 4 University of Southampton, Southampton, UK

**Keywords:** Diseases, Health care, Signs and symptoms

## Abstract

Fractional exhaled nitric oxide (FeNO) testing is used in primary care in some areas of the UK to aid asthma diagnosis but is used less frequently for managing asthma. A randomised controlled trial (RCT) is investigating whether an intervention, including FeNO testing and a clinical algorithm, improves outcomes for patients with asthma. This study was conducted to explore potential for implementation of the intervention. The study aim was to explore views of those with a vested interest in implementing the FeNO intervention into primary care asthma reviews. In-depth, semi-structured interviews were conducted online with individuals, including those with experience in policymaking, healthcare management, National Health Service commissioning, as healthcare professionals (HCPs) with extended roles, and patients and advocates. Inductive thematic analysis was conducted for nineteen interviews. Findings suggest complex interplay of barriers, contextual issues and facilitators. Overall, participants perceived FeNO-informed asthma management would enhance care, if used appropriately and flexibly according to context, for example planning implementation alongside remote reviews. Easier, equitable access to funded FeNO equipment would be needed for national implementation. Participants suggested motivation of all involved in future implementation may be increased by guidelines recommending FeNO, and by use of financial incentives and champions sharing best practice examples. In conclusion, financial obstacles were reiterated as a primary barrier to FeNO use. Despite barriers, facilitating implementation by harnessing prominent cost-benefits could persuade decision makers and clinicians. Findings lay early foundations for development of an implementation strategy.

## Introduction

Asthma is a common condition characterised by symptoms such as shortness of breath, wheeze and chest tightness^1^. Treatment with inhaled corticosteroids (ICS) aims to reduce type-2 inflammation in the airways, thereby reducing risk of future asthma attacks^2^. Asthma control is suboptimal across many higher and lower income countries and contributes significantly to global non-communicable disease burdens^3^. Asthma management and outcomes are suboptimal in the UK, where patients continue to experience asthma attacks and avoidable deaths^4^.

ICS under-prescribing and failure to recognise overuse of short-acting-β_2_-agonist bronchodilators (SABAs) as an indicator of poorly controlled asthma were identified as contributing factors in the UK National Review of Asthma Deaths (NRAD)^4^. These issues were echoed in the global asthma report^3^. Routine asthma reviews are generally conducted annually in UK primary care, to monitor, discuss and manage asthma, and promote patient self-management. However, existing approaches risk over or under-treatment using ICS, since they neglect objective measures of inflammation and often do not provide adequate assessment of risk of asthma attacks^5^. Fractional exhaled Nitric Oxide (FeNO) is an objective measurement of airway inflammation that can be used in patients with asthma^1^, and has potential for refining personalised asthma management through non-invasive testing and a ‘treatable traits’ approach that could be used in primary care^6^. In 2017, the National Institute for Health and Care Excellence (NICE: an independent organisation who evaluate health technology for use in the UK National Health Service (NHS) and provide guidance to healthcare professionals/HCPs), called for evidence on the impact of FeNO testing in improving asthma management^7^. In response, we developed a behavioural intervention using the person-based approach (PBA)^8^, to incorporate FeNO into asthma reviews. The intervention includes the FeNO test and a web-based algorithm giving personalised recommendations for HCPs regarding patients’ asthma management. A variety of webtool recommendations can be made, for example, step-up/down medications, change medication, refer to secondary care, and discuss other management aspects, such as inhaler technique. The intervention also includes online training to use the webtool and a patient information booklet. The DEFINE (Determining the effectiveness of an online FeNO-guided asthma management intervention in primary care) randomised controlled trial (RCT), will test effectiveness and cost-effectiveness of the intervention to reduce risk of acute asthma attacks in primary care, for those over the age of 12 years^9^. If effective, the goal is to implement the intervention across primary care.

Historically, both NICE^7^ and the British Thoracic Society (BTS) in collaboration with Scottish Intercollegiate Guidelines Network (SIGN) produced UK guidance for HCPs managing asthma^10^. In 2024, new joint NICE/BTS/SIGN guidelines suggest FeNO can be considered for monitoring asthma during reviews and before/after changing therapy^11^. This was based upon clinical effectiveness and cost effectiveness evidence reviewed by NICE-BTS-SIGN, identifying that FeNO incorporation provides an additional tool for monitoring and is cost-effective for adults, compared with usual guideline-based monitoring, but not for children^12^. They note that asthma control and quality of life improved with implementation of spirometry and FeNO-guided reviews^12^. International recommendations suggest use of FeNO to monitor children’s asthma, with caveats due to costs and availability^13^. The Global Initiative for Asthma (GINA) report identifies FeNO has a role in supporting medication decisions (particularly for severe asthma), but that effectiveness evidence is heterogeneous and complex^14^. Such heterogeneity limits meta-analysis of effectiveness studies^15^ and much of the effectiveness evidence comes from secondary care studies. However, low FeNO and FeNO-guided management were associated with lower ICS use in a trial (sub-group analysis) of FeNO and symptom-led management for adults in primary care^16^. It is anticipated that the DEFINE trial^9^ results will add clarity to effectiveness and cost-effectiveness evidence^17^.

Qualitative research suggests patients want personalised asthma care, including suitable, reliable and convenient medications, and non-judgmental discussion, empowering them to achieve asthma control^18^. The inclusion of FeNO-testing to inform management has been identified as useful in empowering patients and supporting personalised asthma care, by providing an objective measure to discuss in clinic^19^.

Previous research has reported FeNO testing is feasible in primary care and acceptable to paediatric^20,21^, adult patients^22^ (including pregnant women^23^), and HCPs but that appropriate training and strategies to implement FeNO are needed^21,23^.

Implementation is a complex process and there is often an evidence-practice gap^24^. It is important to understand the diverse factors which could influence adoption (the act of adopting a new practice) and implementation (the process of enacting this new practice)^25^. Previous qualitative work has reported HCPs’ views of using FeNO testing^19,26^ and this will be supplemented by a process evaluation nested within the DEFINE RCT^9^. However, the important perspectives of those potentially implementing or able to influence implementation of the FeNO intervention in primary care asthma reviews have yet to be considered. This study aimed to address this by exploring perceived barriers and facilitators to implementation with this group.

## Methods

### Study design

A qualitative interview study was conducted to explore views and experiences of groups with a vested interest, providing insight to support implementation of the FeNO intervention.

### Advisory group involvement

An advisory group was set up to inform study design and analysis. Advisors were invited from the investigators’ existing networks and had experience of policy development, implementation research, guideline writing, NHS commissioning, managerial roles, professional body board positions, and local and regional leadership* positions (*Integrated Care Boards (ICBs, in the UK context). Eight advisors participated in two meetings. Meetings were either audio-recorded or notes were taken and shared with advisors afterwards to check accuracy. The first meeting sought feedback on the interview topic guide, prior to study recruitment. The second meeting was conducted during recruitment and involved discussion of initial findings and plans for targeted recruitment going forwards, including theoretical sampling to explore areas of interest in greater depth^27,28^. This included both targeting recruitment, for example, clinicians working with children and in areas of high socioeconomic deprivation, and reviewing ongoing analysis for these areas of interest.

### Sampling and recruitment strategy

Eligible individuals were those with a vested interest in the implementation of FeNO testing in primary care, over the age of 18 years, and in a relevant role for at least three months.

Interview participants were purposively sampled via investigators’ networks through individual and professional group email shots, with subsequent snowballing and theoretical sampling^28^. Initially, those involved in decisions about adopting, implementing and, or conducting FeNO testing in primary care were invited, including policymakers, charity representatives, healthcare managers, NHS commissioners, guideline groups, patients, HCPs and industry representatives. We also invited patient-advocates, through existing networks.

Invitations were sent between June 2023 and June 2024. Sixty-three individual invitation emails were sent by the researcher (GL), with one subsequent reminder, as appropriate. Group mailshots were sent by thirteen organisations (for example, the Primary Care Respiratory Society (PCRS), often via administrators, therefore it is not possible to quantify total numbers invited. Those invited to participate in advisory meetings and interviews were asked to forward invitations to their networks.

### Interviews and data collection

Participants consented via an online form or verbally prior to interview. Verbal consent was recorded by the interviewer (GL) on a consent form and a copy was sent by secure email to participants.

Data were generated through online semi-structured interviews using Microsoft Teams. A copy of the initial interview topic guide is available [Supplementary Information]; topics broadly explored experiences with implementing new practice, including FeNO where relevant; perceived barriers and facilitators; how FeNO inclusion may or may not align with review priorities and how engagement with implementation may be influenced. This was tailored according to participants’ roles and refined according to ongoing findings. Questions were open to allow the interviewer to be guided by participants’ discussion, whilst using the topic guide to ensure coverage of topics. Prior to interview, participants were provided with access to the FeNO-guided webtool and online training, to allow them to complete the training (lasting approximately 30 min) and see the webtool functionality. However, many could not commit to looking at this ahead of interviews. In these cases, pre-prepared slides explaining the intervention and a short video demonstrating the web-based tool were shown. Patients were sent or shown patient-facing resources. In cases where patients were unaware of what the FeNO test involves, a demonstration video was shown.

Interviews were audio-recorded, transcribed by an independent transcriber, checked, and pseudonymised. Participants were provided with shopping vouchers to reimburse their time taken to participate. Data analysis and collection were concurrent. Recruitment ceased when the team and advisors determined that data were sufficiently rich and in-depth to answer the research question^29^.

### Data analysis

De-identified transcripts and field notes were repeatedly read to aid familiarity and memos were made throughout, attending to reflexivity and providing a record of analytic decision-making^30^. Those collecting (GL) and analysing data (GL, MS, BA, STC)) included a mix of those who had been involved in previous work regarding FeNO inclusion in asthma reviews (MS, BA, STC) and those who had not (GL), and all have experience of qualitative research, particularly related to asthma, intervention development, testing, refinement and implementation.

Inductive thematic analysis^31,32^ was iteratively conducted, including coding, development of candidate themes and theme refinement, and team discussion. The second advisory meeting occurred during early analysis and allowed discussion of developing findings with experts in the field, and suggestions for theoretical sampling to address perceived gaps in the sample that may permit more transferrable findings.

## Results

### Participant characteristics

Nineteen individuals participated in interviews. Participants’ extended roles and reason for inclusion are shown in Table 1. The sample included 6 general practitioners (GPs), 4 nurses, 3 pharmacists, 1 respiratory physiologist and 5 patient advocates or patients with experience of asthma and patient and public involvement (PPI) responsibilities. Ten participants used FeNO in primary care, including community diagnostic centres (CDCs) in two cases. Whilst participants were recruited due to their extended roles (e.g., guideline writing) we identify participants by their clinical role or as patients, alongside quotations, to provide context, whilst preserving anonymity. Further participant details are reported in Table 2. Online interviews occurred between June 2023 and May 2024, and lasted between 32 and 54 min (excluding time to explain the intervention).

**Table 1:** Interview participants’ relevant roles and duties.

Relevant roles or duties (at any point in careers)	Number of participants reporting having held these roles/had experience* in these areas (*not limited to)
Experience with piloting and /or adopting and implementing new innovations (including FeNO for respiratory diagnoses)	15 Of those 15, 11 also had experience with piloting/adopting and implementing FeNO testing in primary care.
Experience contributing to asthma guidelines and/or policy	4
Regional, or local respiratory leadership in primary care	6
Commissioning in primary care	1
Managerial roles in pharmacy, NHS and/or primary care	6
ICB leadership roles	2
Professional body board positions and contribution to guideline writing (e.g. nursing groups)	8
Charity support roles and advisory roles	2

**Table 2:** Interview participants’ experience and institutional affiliations.

Participant characteristics	Number of participants in each characteristic category
Duration in current role	
Less than 1 year	0
1–2 years	1
2–5 years	1
5–10 years	2
10 years or more	9
*Not disclosed*	6
Clinical role working with patients	
Yes	13
No	6
Organisations they work/volunteer for (there may be more than one for each participant)	
NHS	14
Academic institutions	8
Charity	6

Two themes were identified:

(1) The impact of the primary care context on FeNO implementation and (2) supporting understanding of the role of FeNO-informed management to increase motivation.

### Theme 1: The impact of the primary care context on FeNO implementation

Participants were mostly positive about FeNO testing and the intervention. However, several contextual issues were raised. Participants talked about how local ‘team cultures’ have different approaches to adoption and implementation of innovation. Local decision-makers were perceived as either cost-focused or patient benefit-focused, which were seen as competing demands:

‘Yes QOF* *is essential, but we’re very much a quality focussed PCN. Some are budget centred.’* (Nurse4)

*QOF: Quality and Outcomes Framework: a reward and incentive scheme used in primary care NHS England to provide resourcing and reward good practice^33^.

A broad, national sense of ‘*discontent*’ amongst HCPs, due to under-funding and increased workloads in primary care, was raised by participants. This was felt to impact the time available to apply for additional funding to introduce innovation, which is often done in HCPs’ own time, therefore requiring significant commitment. Conducting FENO-informed asthma management was also viewed as adding to workload pressures:

*‘Practices are conservative – with a small ‘c’ – in terms of resistant to change. It’s not even resistance. It’s just a passive inertia. You’ve got to give them a really good reason to want to make the leap into things they don’t have to do.’* (GP6)

Participants reported several factors that have created a challenging context within which to implement innovation into asthma reviews. Limited numbers of HCPs in primary care with additional training and expertise in asthma was perceived as limiting scalability of a FeNO intervention:

*‘Less than 5% of the nurses and other HCPs caring for patients with asthma in primary care have any training whatsoever in asthma*.’ (GP2)

This was echoed in patients’ accounts of varied asthma review quality and provision across practices:

*‘I think I’ve had six different GPs in* (area) *and* (area)*. Each one managed asthma differently. Asthma reviews - either didn’t do them. All* (HCPs) *did it in a different way. So, it’s very difficult to have that approach.’* (Patient/advocate3)

Participants stressed that online training for the webtool was acceptable but needed to be supported by hands-on training for FeNO-testing, with transparency over costs and time needed to train staff. However, concerns about using electronic systems that are not interoperable with current systems were noted, partly due to the time demands of using numerous systems:

*‘I deal with 165 long term conditions. I don’t want to have to go to 165 different web-based tools. They must be integrated in my clinical system otherwise I’m wasting time.’* (GP3)

However, some participants reported accepting use of different systems as standard.

Having a flexible approach to implementing FeNO and the intervention was seen as vital to keep up with evolving practice, such as provision of remote reviews. HCPs explained that having high numbers of students and commuters as patients meant virtual reviews were preferred, whereas HCPs working with populations in deprived areas highlighted the need to engage directly with patients to explain the need for reviews and conduct them face-to-face:

‘(in) *many of the deprived areas, there is an issue of health literacy. One of the reasons why my clinic was successful was that I called patients, highlighting the benefits of the service and therefore the use of technology.’* (Pharmacist3)

Patient-facing participants were asked about perceptions and experiences of implementing FeNO-informed reviews with children/young people (CYP). Whilst all described conducting asthma reviews with CYP and adults, they did not identify specific differences in implementation (noting the intervention is designed for those over 12 years-old). One participant highlighted that understanding instructions is a key factor, rather than age, and that FeNO machines must be set correctly for children:

*‘It all depends on the individual child, whether they’re able to understand and whether they can perform the test…. And ensure the FeNO machine is set to child mode, because if they don’t change it to the child setting then the children may not have the capacity to blow to completion and so the tests will be void.’* (Pharmacist2)

Implementing the intervention into existing clinic time slots was also seen as a barrier for HCPs, who are already affected by suboptimal scheduling:

*‘I think for a good asthma review*, (you need) *30* *min, but in surgeries I know, nurses usually get 20* *minutes. I’ve seen some 15* *minute slots.’* (Pharmacist1)

Upskilling support staff was seen as an option to reduce concerns about staff costs, time and capacity to include FeNO-testing:

*‘It could still be more cost effective because a healthcare assistant* (HCA) *rather than a trained respiratory nurse can do FeNO. Patients could have FeNO and then a telephone or virtual review with a respiratory nurse’* (GP5)

Some participants highlighted current challenges around performing the test in one location (CDCs/pharmacies) and sending results to HCPs elsewhere, without supportive decision-aids:

*‘I have to pass back* (the FeNO result) *to the clinician to decide because it is their patient. What do I do then? I mean how do we then train the clinicians in understanding the results and perhaps doing something, so it’s not just about us doing it.’* (Pharmacist1)

Additionally, such two-step appointments may be burdensome for patients, may increase the likelihood of missed appointments, and further work to invite patients for repeat appointments. The chance to immediately discuss FeNO results, was seen as an opportune ‘*teachable moment*’ particularly for encouraging optimal treatment adherence. Therefore, one appointment including FeNO testing with one HCP was suggested as gold standard:

*‘FeNO would really support conversations that help support self-management. I wouldn’t go without using FeNO in my respiratory clinics.’* (Nurse4)

Patients also recognised they may gain more from early and repeated FeNO-testing, with easier, local access in primary care, since FeNO results supported understanding the need to manage triggers, particularly where allergen sensitivity is present:

(having ‘*allergic asthma’* and) *‘having that test in primary care, without all that extra travel, all that extra waiting time as well, I think would be great.’* (Patient/advocate4)

Local and national issues will affect implementation; therefore, implementation strategies must reflect flexible practice and population needs.

### Theme 2: Supporting understanding of the role of FeNO-informed management to increase motivation

Participants recognised that motivation of both key decision makers and HCPs is needed if FeNO-informed asthma management is to be implemented successfully.

Participants reported asthma is under-prioritised by decision-makers compared with many long-term conditions, despite high prevalence, significant impact on patients and NHS costs. Participants recommended persuading decision-makers by highlighting cost-savings and benefits linking with top-level government policy agendas:

*‘We had the National Review in Asthma Deaths* (NRAD), *we’re doing worse than before. Asthma deaths are not sexy enough…… the first time ever I’ve heard practices sound almost interested in respiratory was because they were incentivised by the Impact Investment Fund, but the hook was the green agenda.’* (GP4)

Participants felt that not having a national drive, or government health aim towards asthma innovation meant that access to interventions was inequitable:

*‘All of a sudden it’s being researched in* (city), *and they’ve got loads of FeNO machines and you wonder whether it’s who you know, and I think that’s a little bit sad if I’m honest’* (Nurse3)

HCPs were positive about use to help consider co-morbidities and optimise treatments and adherence and suggested communicating benefits could motivate uptake, but expressed concerns that patients may not accept treatment changes if they do not recognise they have sub-optimal asthma control:

*‘FeNO tests help us distinguish patients who are having symptoms because they are anxious and it’s a breathing pattern disorder or laryngeal dysfunction or you have non-adherence. …A patient barrier… when they are used to being breathless, and they have high FeNO, they feel that’s my normal’* (GP1)

Patients were critical about the usefulness of other tests, such as peak flow, and believed FeNO was more relevant to day-to-day symptoms and planning management:

*‘They* (peak flow/Spirometry) *don’t always indicate how you feel, whereas I’ve always found FeNO to be a lot more sensitive’* (Patient/advocate5)

Patients were in favour of FeNO to optimise management and noted acceptability of management changes depend upon good communication:

*‘FeNO, gives a good indication, especially when you’re on high level steroids, to say ‘actually it does look like the inflammation particularly in your lungs, is dampened down. We can try reducing this medication now’, which obviously…no one wants to be stuck on steroids, so* (if) *you can reduce them safely, it’s great.’* (Patient/advocate2)

HCPs with access to FeNO machines and training provision (through Academic Health Science Network (AHSN^34^) reported benefits of sharing best practice for both their learning and encouraging others to implement FeNO. However, they flagged that where programmes were discontinued, practices had to cover costs for consumables, if they wished to continue use, which was not always possible, even when motivated.

*‘NHS England funded a lot of FeNO testing. They gave some money to AHSN to try and implement that which has now discontinued.’* (GP3)

Discontinuation of funding consumables and insufficient time allocation led to HCPs being selective over which patients had FeNO testing, based on their own judgments. This was viewed as reasonable where HCPs are experienced and well-trained in managing asthma, but unacceptable as a widespread approach.

Two participants described receiving training from FeNO machine manufacturing representatives. One participant described feeling unsupported in accessing training, whilst being expected to lead FeNO implementation:

*‘I’m leading this* (CDC) *hub. I’m starting out with looking at all the protocols…starting from scratch. There’s nothing at the moment* (to expand workforce training) *and so I’m just adding things to it. I’ve got to bring FeNO in very quickly.’* (Pharmacist1)

Those already using FeNO in practice reported a transition from cynic to convert, over time, and suggested clinic-based teaching sessions would motivate implementation:

*‘I went from being quite cynical about FeNO to…a light bulb moment where I realised this was specifically looking at inflammation.’* (Nurse1)

Where financial incentives might also motivate implementation, the QOF, for example, was also seen as promoting an insufficiently personalised *‘tick-box’* review culture.

Motivating through increasing understanding of FeNO could address concerns related to misunderstanding the role of FeNO as purely a diagnostic test, or a test for use in secondary care:

*‘It’s quite a simple test – I don’t think people understand the test, it’s sold as a test for diagnosing asthma – they just think, ‘Oh, no–goodness, here’s another thing we’re being expected to do.’* (Patient/advocate1)

Advocates or champions were suggested as useful in persuading HCPs of intervention benefits. However, participants raised that a delicate balance is needed to persuade HCPs to use a decision-aid such as the webtool, because they wish to retain clinical autonomy and responsibility and ensure that all components of their asthma review are considered, to avoid concerns about *‘overreliance on the test’* (GP6).

This balance was felt to be further complicated by the varied levels of experience and expertise in managing asthma amongst those delivering reviews in primary care. Participants noted experienced HCPs may underutilise the web-tool due to their feeling confident and qualified to interpret FeNO without a decision-aid.

Yet, if used by less experienced staff, some believed the web-tool would address fears about stepping-down medications and make practice *‘safer’* (Nurse2) for those with less experience in interpreting FeNO results, and partially address the shortfall in experienced staff available to conduct asthma reviews, provided there is evidence to support use:

*‘You need data to show that it’s going to reduce attacks, and it’ll do so with the equipment in the hands of somebody who is not an asthma expert’ (GP2)*

Since many participants believed only some patients need to have FeNO-testing, guidelines and robust evidence are needed to guide patient selection. Yet, this is complicated by differences between national and international guidelines and varied adoption of them in practice:

*‘Well, the UK guidelines are on average three to five years out of date at any time that you look at them…..GINA updates it’s guidance document every year.’* (GP2)

‘*NICE and BTS-SIGN guidance had two opposing views* (regarding FeNO) *and so what they’re gonna have to do is work out a compromise based on the evidence and the cost economics.’* (GP3)

Participants believed guidelines can motivate implementation but that guidelines for FeNO are not explicit for monitoring and more evidence is needed to allow guideline writers to promote implementation.

Where guidelines are changed, participants raised concerns that implementation strategies must also allow time and planning for implementation into practice, as unexpectedly requesting change is demotivating for HCPs:

*‘Where new guidelines come out, we’ve been asked to suddenly fit something into a service, obviously staffing, training, rooms, was very difficult’* (Respiratory Physiologist)

Aside from contextual practicalities and the need for motivation, participants were unanimous on the importance of striving to prioritise improving asthma care and outcomes, and FeNO-informed care supported these goals. Participants highlighted the importance of patient inclusion in implementation strategy development, alongside other groups.

## Discussion

Participants identified contextual issues and interplaying barriers and facilitators likely to affect adoption and implementation of FeNO-informed asthma reviews. Funding was repeated as a major obstacle, and participants noted that (national) decision-makers must be convinced of the benefits of FeNO. Contextual issues raised included considering local patient population-needs, access to HCPs with an asthma qualification, training in FeNO-testing for HCPs, and allocated time to incorporate FeNO measurements and clinical algorithms into asthma reviews. Broader issues influence the likelihood of FeNO implementation, such as, current under-prioritisation of asthma, evolving guidelines, and current inequitable access to FeNO machines. Those with an existing interest in respiratory medicine are likely motivated to implement innovation that has been shown to improve outcomes, whereas others may require different approaches to encourage them to implement the intervention.

To our knowledge, this is the first study to explore perceptions of the implementation of FeNO-informed asthma reviews in primary care, with those with a vested interest and potentially, the ability to influence decision-makers. Advisor consultation strengthened several iterations of codes, categories and themes, and discussions heightened reflexivity.

Despite an elongated recruitment period, a deliberately broad eligibility criteria and varied sampling techniques, we were unable to reach some target groups for interview (e.g. charity representatives). However, some of these were represented in the advisory group who fed into the study design and analysis. Many of our patient-facing participants had experience of using FeNO. This is a strength because they have direct experience of barriers and facilitators to implementation. However, having fewer participants without experience of FeNO may mean other factors were not considered and may limit transferability of the findings, particularly since the recent NICE-BTS-SIGN guideline committee noted that FeNO is not yet widely used or accessible in the UK^12^. Inclusion of patients, PPI representatives and patient advocates is a strength, particularly, as it has been reported that patient ‘*factors*’ remain under-researched in considering implementation of digital interventions^35^. Our advisory group were especially interested in patients’ perspectives on implementation of the intervention. This work, alongside exploring HCP views, will be enhanced by a process evaluation of the DEFINE RCT.

The findings are broadly in line with studies examining barriers and facilitators for implementing innovation in health-settings, particularly financial costs, lack of wider political support, training, motivating and supporting the workforce^36^. Our study describes the nuances involved in asthma reviews, and aligns with other reports of a reducing workforce but increasing demands^37,38^. Our interviewees reported significant funding concerns, at least partially attributed to under-prioritisation of asthma, which is noted elsewhere^39^. Findings support evidence that patients and HCPs believe FeNO adds value to asthma reviews, particularly in enhancing patient education and empowerment, but that expense precludes use and interpreting results can be challenging^19^ without supportive decision-aids. Whilst our study is influenced by local contextual factors, some may be transferrable to other contexts, for example, our participants noted FeNO supports adherence discussions. It is recognised that suboptimal medication adherence is likely a global issue, even where excellent healthcare is available^3^. Innovative approaches to improve this and standardise national asthma care are recommended^3^.

Champions were considered useful in motivating and influencing implementation. This has been suggested in other qualitative studies exploring implementation of innovation in asthma reviews^40^. However, evidence also suggests champions’ roles are varied and broad in scope, and further research is needed to understand how champions effectively influence implementation^41^.

Implementation strategies must be flexible, due to changing contexts^42^. Since our study interviews, NICE-BTS-SIGN produced joint asthma guidelines, suggesting considering use of FeNO to monitor asthma during reviews and before/after changing therapy^11^. NICE previously included FeNO use for asthma diagnosis in their guidelines^7^, yet inequitable access to care is a continued challenge^43^ and not all practices have a FeNO machine^12^. Whilst our participants suggest having FeNO-use promoted in guidelines should help implementation, they noted this must be nationally supported.

Our results suggest complex interaction between barriers, facilitators and contextual influences, whereby employing facilitators can introduce new barriers. For example, participants suggested well-trained HCAs could conduct FeNO testing (HCAs work in support roles in the UK, and often take on additional clinical duties). Whilst this might alleviate cost and time pressures on HCPs, it may mean loss of the teachable moment in explaining FeNO result implications to patients. Similar concerns were raised by those performing tests in CDCs. Given CDCs have become more prevalent and have FeNO-testing capability^44^, this is an important consideration that could be further examined. Such complex interactions have been noted elsewhere when implementing electronic or digital intervention^45^ and warrant consideration in implementation strategies. Moreover, literature reporting advantages, disadvantages and nuances involved in supported self-management^46^, including using remote delivery^47^ and e-health^48^, should be considered alongside our findings, since introducing FeNO-testing would necessitate patient attendance, at least for the FeNO-test. Our interviewees largely believed FeNO inclusion would enhance supported self-management, and recommended integrating training into existing asthma training packages, including hands-on approaches. We suggest future implementation studies explore this and the use of champions and how these may influence implementation across diverse primary care settings.

Future work could use the PBA^8^ to both refine the intervention, considering practical issues raised, such as maximising webtool system interoperability, and to co-develop an implementation strategy with advisors. Implementation strategies have been defined as the ‘*how to’* component of changing healthcare practice^49^, and it is likely that the strategy will need to be tailored considering evolving evidence, guidelines and our findings. Research could also explore implementation for CYP in greater detail, from perspectives of clinicians, CYP and their parents/guardians.

In conclusion, financial support for adoption, implementation, sustained use and coverage of consumables will likely be necessary. Even where funded or incentivised financially, clinicians highlighted the need for time to train and adequate time for asthma reviews in clinic. Motivating clinicians may be difficult given contextual pressures, but champions sharing latest evidence and experiences are likely to be influential and impactful.

## Supplementary information

Supplementary Information

## Data Availability

The data that support the findings of this study may be made available upon reasonable request to the corresponding author.
