# Supplementary material for: Qualitative insights into planning implementation of FeNO-guided asthma management in primary care
Source: NPJ Prim Care Respir Med. 2025 Mar 20;35:16. doi: 10.1038/s41533-025-00418-w (PMC11926368; doi:10.1038/s41533-025-00418-w)
Supplement: Supplementary file 1 — Supplementary Information [file 41533_2025_418_MOESM1_ESM.docx]

**Exploring the implementation of FeNO testing and a FeNO-guided algorithm in primary care**

**Interview Topic Guide**

## Briefing

1. Welcome and thanks to participant for agreeing to take part.
2. Introduce self.
3. We would like to talk about how you think it would be best to introduce FeNO testing and a FeNO guided algorithm in primary care, and any issues you can think of with this. Differences between people’s views may come about from different perceptions of settings and organisations, from different priorities, and from different beliefs about healthcare provision. These differences are important to us, and we value your unique perspective.
4. If at any time you do not wish to answer a question, that’s okay.
5. I would like to audio record our conversation. It will be deleted once the data have been analysed. The recording will be written up as a transcript, but it will be labelled with an ID number that does not identify you. Your name and any names you mention, and any places you mention will be taken out, so that if someone read the transcript, they would not know who you are or where you work.
6. Your participation will remain confidential.
7. If, at any stage, you wish to pause or stop the audio recording, please let me know.
8. Do you have any questions before we start?

## Topics to be explored

Below is a list of topics to be discussed. The topic guide will remain flexible with respect to what is of importance to participants, however, the key topic of stakeholders’ views and experiences of introducing FeNO testing to primary care will remain the same.

A couple of PowerPoint slides showing the FeNO-guided algorithm will be shared with participants in advance to help them consider how this would work in practice if they are not already familiar with it from the DEFINE feasibility study or randomised controlled trial. Once a preliminary version of the implementation strategy and materials have been developed, this will also be shared in advance with participants.

1. Participant views on their organisation and its role in introducing/implementing FeNO testing and a FeNO-guided algorithm in primary care for the management of asthma.
2. Participant views or experience of existing processes, roles and strategies used within and across different organisations when introducing new approaches to primary care consultations.
3. Participant views or experience of (potential) barriers and facilitators to the adoption of FeNO testing and a FeNO-guided algorithm in primary care for the management of asthma.

**Example questions** (additional questions may be added following the topics above):

1. Can you briefly describe your position and role within your organization?

Prompts: What are your main responsibilities? How does your role link to asthma reviews in primary care?

1. Can you briefly describe the main goals and main activities of your organization?

Prompts: What type of organisation do you work in? How is it funded? Which other organisations do you have links with?

1. Can you describe the area you work in and its population? (prompt: for example, is it largely an affluent area or an area of high social deprivation?)

We are interested in supporting the adoption and sustained use of FeNO testing and a FeNO guided algorithm in asthma reviews to help improve asthma management. If you could bring up the PowerPoint slides I sent about FeNO testing, we can go over what this would look like?

[Talk through slides and ask if they have any questions]

1. What do you think about introducing FeNO testing and a FeNO guided algorithm in asthma reviews?

Prompts:

Do you have experience of testing FeNO in primary care? (If so, can you talk about that experience?) /Have you had training for this? / If not, what do you think of the idea of it?

Are there any examples where you think this would work particularly well/not well?

What do you like about this plan?

What do you not like about it?

What would you change?

Who else should be involved?

What else should be done?

How feasible do you think this plan is?

How could we put this into action?

1. In your experience, what approaches have worked best when trying to implement new technology or new approaches in primary care?

Prompts: Which types of policies or recommendations are most effective? What are the best ways of communicating new changes? Can you give me a good example of something that has been well implemented? Why do you think this was this successful? What can we learn from these examples?

1. What approaches have not worked well when implementing new technology or practice approach?

Prompts: Can you think of an example of something that was not well implemented? Why have these been unsuccessful? What can we learn from these experiences?

Outputs

1. How do you think the online training and digital algorithm could best be provided in practice? What would make it easier to access and use the FeNO guided intervention where you are based?

Buy-in and engagement

1. Who do you think could influence whether this FeNO-guided intervention is successfully rolled out or not in Primary Care?

Further prompts: Who would need to agree to it? Who might make it harder to implement?

1. Who would make a good champion to support and drive the roll-out of the FeNO-guided intervention in Primary Care?
2. Who else should we speak to during this research study to help develop a plan for rolling out the FeNO-guided algorithm?

Further prompts: How might we best involve them?

Fit with system

1. How do you think this FeNO-guided intervention fits in with normal work/asthma reviews/asthma management?
   1. What things within your practice/organisation/experience might influence whether this intervention gets rolled out or not?
   2. What support could help get it rolled out? Are there any changes to current ways of doing things that would be needed? Who can make sure that happens?
   3. What challenges might there be to successfully rolling out the FeNO-guided intervention? How might it change existing practices? How might it need adapting to fit with your practice?

Alignment with priorities

1. What are your priorities for asthma reviews?
2. Do you think a change is needed in how asthma reviews are done? Is there anything that currently doesn’t work well?
3. How might the FeNO guided intervention help clinicians/patients/others?
4. To what extent do you think the intervention is in line with NHS priorities? And what about local priorities?

Outputs and impact

1. What do you think would be important to measure to see if this intervention has made a difference? How soon do you think you would see a difference?
2. Do you think there might be any other effects of using this intervention that we should look out for?

Adoption and spread

1. What might influence whether your organisation adopts this intervention?
2. What might influence other organisations’ adoption of this intervention?
3. How can we make it easier for organisations to adopt it?

How do you think the *sustained use* of FeNO testing in asthma reviews, with or without the FeNO guided algorithm, could best be supported over time? Prompts: Do you in your role, or your organisation, have a role to support this? What are potential barriers and challenges to this process (political, financial, health care system, local manages/governance, available resources, clinicians, patients)?

(Added post 2^nd^ stakeholder meeting) – are there any other contextual issues, for example in areas with high levels of social deprivation, and/or specific populations (e.g., children) that we should consider when planning to implement this intervention?

Concluding questions:

● Do you have any additional remarks?

● Is there something that you think we didn’t cover that is relevant to this issue / topic?

● Is there someone else you think we should talk to, that you can identify as a key stakeholder in this area?

Thank you for your time.
